# Non-contact, synchronous dynamic measurement of respiratory rate and heart rate based on dual sensitive regions

**DOI:** 10.1186/s12938-016-0300-0

**Published:** 2017-01-17

**Authors:** Bing Wei, Xuan He, Chao Zhang, Xiaopei Wu

**Affiliations:** 1 School of Computer Science and Technology, Anhui University, Hefei, 230601 China; 2 Department of Computer Science and Technology, Hefei Normal College, Hefei, 230061 China

**Keywords:** Respiratory rate, Motion artifact, BSS, SOBI, Power spectrum kurtosis

## Abstract

**Background:**

Currently, many imaging photoplethysmography (IPPG) researches have reported non-contact measurements of physiological parameters, such as heart rate (HR), respiratory rate (RR), etc. However, it is accepted that only HR measurement has been mature for applications, and other estimations are relatively incapable for reliable applications. Thus, it is worth keeping on persistent studies. Besides, there are some issues commonly involved in these approaches need to be explored further. For example, motion artifact attenuation, an intractable problem, which is being attempted to be resolved by sophisticated video tracking and detection algorithms.

**Methods:**

This paper proposed a blind source separation-based method that could synchronously measure RR and HR in non-contact way. A dual region of interest on facial video image was selected to yield 6-channels Red/Green/Blue signals. By applying Second-Order Blind Identification algorithm to those signals generated above, we obtained 6-channels outputs that contain blood volume pulse (BVP) and respiratory motion artifact. We defined this motion artifact as respiratory signal (RS). For the automatic selections of the RS and BVP among these outputs, we devised a kurtosis-based identification strategy, which guarantees the dynamic RR and HR monitoring available.

**Results:**

The experimental results indicated that, the estimation by the proposed method has an impressive performance compared with the measurement of the commercial medical sensors.

**Conclusions:**

The proposed method achieved dynamic measurement of RR and HR, and the extension and revision of it may have the potentials for more physiological signs detection, such as heart rate variability, eye blinking, nose wrinkling, yawn, as well as other muscular movements. Thus, it might provide a promising approach for IPPG-based applications such as emotion computation and fatigue detection, etc.

**Electronic supplementary material:**

The online version of this article (doi:10.1186/s12938-016-0300-0) contains supplementary material, which is available to authorized users.

## Background

Currently, IPPG technique has been developed for measurements of physiological parameters, such as HR, RR, etc., which uses an imaging devices to capture video of body surface that contains physiological information, and restructures the information by specific algorithms [1–5]. It has many advantages in physiological parameters assessments, such as low-cost, non-contact, safe, continuous measurement, etc. Hence, IPPG techniques, especially the ones based on ordinary camera and ambient light, have become a research focus in biomedical engineering field [1–18].

Previously, Takano and Ohta initially presented the feasibility of HR assessment based on IPPG system that used ambient light as the illumination [6]. Also, using ambient light, Verkruysse et al. introduced a spatial ROI averaging on R/G/B channels approach, which significantly improved the signal-to-noise ratio (SNR) in IPPG signals [7]. Moreover, they also brought insight into the relative strengths of IPPG signals in different channels, revealed that the G channel carried the stronger BVP signals [7]. Since then, several teams have attended to cardiac pulse researches related to G channel [8–11]. Although G channel suits for HR estimations, the motion artifacts inescapable in IPPG might make the accuracy vulnerable and limit its capabilities in real-world measurements environments [12]. Based upon previous research results, Poh et al. proposed a novel IPPG method based upon Independent Component Analysis (ICA). Using joint approximate diagonalization of eigenmatrices (JADE) algorithm, they separated out the BVP source signal and motion artifacts from the R/G/B channels [13, 14]. As a potential tool, ICA/BSS has the advantages to improve the estimation accuracy of BVP signal along with motion artifact attenuation. Therefore, the approach proposed by Poh et al. has aroused much interests [15–19].

Currently, there have been many IPPG techniques based on ambient light on the theme of how to extract physiological parameters, such as HR, RR, HRV, SpO_2_, etc. [1–18]. However, it is accepted that only HR measurement has been mature for applications, other estimations are relatively incapable for reliable applications. For instance, there are several researches have made short mentions of RR estimation: mainly estimation from spectral peak of the signals generated from video frames based on relevant ROI [3, 6, 7, 20, 21] or estimation from HRV using a well-known indirect method [13, 22], while the sensitiveness to motion artifacts in these estimations has not been carefully addressed [23]. Thus, it is worth keeping on further exploration in RR measurement, as well as other vital signs. In addition, there are still some issues commonly involved in existed approaches need to be optimized and explored further for more reliable and practical measurement in IPPG techniques. Brief analyses are as follows:

### a. Motion artifacts attenuation

It is known that motion artifacts are difficult to avoid in IPPG systems [23]. Take, for example, the IPPG techniques based on facial videos that are most highly concerned. Apart from involuntary global motions such as head swing and deflection, natural motions in local facial regions or even other more complex artifacts should be included in motion artifacts. Commonly, to attenuate motion artifacts, a series of video tracking and detection algorithms are utilized to locate the face in consecutive frames with a rectangular bounding-box, such as the Viola-Jones (VJ) face detector [13]. By simply employing these video tools, one can only compensate for the global motion of the whole face, without capability to cover more. Among different local regions on face, there are significant differences on SNR. For instance, the regions of cheek and forehead are golden for assessments, while the regions of eyes, nose, mouse are ill-suited, which invariably arise local motion artifacts like blinking, wrinkling nose, yawn, as well as muscular movements caused by smiling, talking, or breathing. This issue is always intractable for studies in IPPG, and has been mentioned and explored by several researches. Wang et al., pointed out limitation of VJ detector, introduced a “tracking-by-detection” with kernels method which is superior than other tracking algorithms and a skin/nonskin pixel classification method for achieving high SNR pulse signals [24, 25]. Feng et al. selected two golden ROIs on the region of cheek that has a higher SNR for assessment instead of the whole face, utilizing a speeded-up robust features (SURF) detector [26]. Emrah Tasli mentioned shortages in traditional tracking algorithms, and proposed a facial landmark localization method to track golden ROIs for obtaining robust signals [27]. Mayank Kumar also mentioned the similar issue, and introduced a new method for generating high SNR PPG signals from the tracked golden ROIs structured by a weighted average approach [28]. Generally, these researches mainly focused on obtaining high SNR signals by tracking selected golden ROIs using different sophisticated facial video tracking and detection algorithms. Attributed to the complicated facial physiological structure and the complex interaction of light with facial tissues, or even weak ambient light changes, the motion artifacts and other complex noises could hardly be attenuated thoroughly by only employing video approaches for local golden ROIs. Moreover, the computational complexities of the video algorithms should also be taken into account for applications of IPPG on different platforms. Considering the capabilities of the ICA/BSS approaches in separation of BVP source signal and motion artifacts, it would be a quite appropriate solution for motion artifacts attenuation in IPPG. Furthermore, some motion artifacts could be deemed as vital signs, such as respiratory motion artifact which contains stable breathing rhythm, it might provide new insights into physiological parameters assessments.

### b. The issues unresolved in ICA/BSS-based IPPG techniques

Most of the existing studies on ICA/BSS-based IPPG techniques are similar to the researches from the method proposed by Poh et al. [13, 14], with seldom further exploration. (1) *Limitation of ICA/BSS based on single ROI* According to the theory of ICA/BSS, insufficient observations would influence the effect of separation. Based upon single ROI (3-channels R/G/B signals), the method proposed by Poh et al. is only fit for single target extraction (BVP signal) with limited motion artifacts attenuation [14], thus it needs to increase the number of R/G/B channels for improvement of separation. Estepp et al. introduced a novel BSS-based method, which employed nine synchronized cameras to capture multiple imager channels, and separated out satisfactory BVP signal with motion artifacts mitigation [29, 30]. (2) *Selection of BSS algorithm* Commonly, the JADE or FastICA, are utilized for extracting BVP signals [14, 17–19]. Among different ICA/BSS algorithms, there invariably existed significant differences in computational complexities, as well as performances of separation, which are both crucial for applications of IPPG techniques. Thus it deserves to select an appropriate algorithm that could maintain the balance between these two points [23]. (3) *Permutation problem of ICA/BSS* There is an inherent permutation problem in ICA/BSS, i.e., the outputs of separation are in random order, which would bring trouble for identifying the target. Poh et al. selected BVP signal only depending on experience alone (selected the second one) [14] and the highest spectra peaks of ICs [13]. Many other studies also employed the similar means [31–33]. In general ICA-based IPPG, for single BVP signal identification from 3-channels outputs, these means could cover it basically. However, when selecting multiple targets from more outputs, this problem would become more complex, and should be highlighted.

In this study, in order to realize the synchronous detection of RS and BVP signal, we explored the potential of dual-region-based BSS method. Two sensitive regions corresponding to RS and BVP detection were selected based upon experimental analysis. Since the two facial regions can yield 6-channel R/G/B signals, it allows BSS algorithms work more stable and efficient in separating multiple physiological signals. It has to be mentioned that we took respiratory motion artifacts for extracting RS. In addition, kurtosis-based identification methods were proposed to solve the permutation problem of BSS, which is crucial for long-term RR and HR monitoring.

## Theories

We first introduce the relevant theories involved in the proposed method, including generation of R/G/B signals from video of body surface and BSS algorithm.

### Theory of R/G/B signals generation

We generated R/G/B signals by spatial ROI averaging, a simple approach that is commonly used in relative studies. Of note, in order to control computational complexity, the proposed method has not employed video tracking tools, such as VJ detector, to compensate for the global motion of the whole face. Here we briefly give out calculation formula and variable symbols that involved in the following sections. Assume R/G/B components have the expression:

1 $$\varvec{I}(x,y,t) = \left\{ {\varvec{I}_{\varvec{R}} (x,y,t),\varvec{I}_{\varvec{G}} (x,y,t),\varvec{I}_{\varvec{B}} (x,y,t)} \right\};\quad 1 \le x \le N,1 \le y \le M$$

where $N$ and $M$ are the height and width of the selected ROI. Then R/G/B signals denoted by $\overline{{\varvec{X}_{\varvec{V}} }} (t)$ are calculated as follows:

2 $$\overline{{\varvec{X}_{\varvec{V}} }} (t) = \left\{ {\begin{array}{*{20}c} {\varvec{x}_{\varvec{R}} (t) = \frac{1}{N \cdot M}\sum {\varvec{I}_{R} \left( {x,y,t} \right)} } \\ {\varvec{x}_{\varvec{G}} (t) = \frac{1}{N \cdot M}\sum {\varvec{I}_{G} \left( {x,y,t} \right)} } \\ {\varvec{x}_{\varvec{B}} (t) = \frac{1}{N \cdot M}\sum {\varvec{I}_{B} \left( {x,y,t} \right)} } \\ \end{array} } \right\};\quad \begin{array}{*{20}c} {1 \le x \le N,1 \le y \le M,} \\ {t = 1,2, \ldots ,T} \\ \end{array}$$

where $\varvec{x}_{\varvec{R}} (t)$, $\varvec{x}_{\varvec{G}} (t)$ and $\varvec{x}_{\varvec{B}} (t)$ are R, G and B component mean values respectively, and $T$ is the number of the frames in sliding window.

### Theory of BSS

Blind source separation (BSS) refers to the method that uncovers hidden source signals from observed signals in the case that the source signals and parameters of transmission channels are unknown, only according to the statistical characteristics of the source signals. Assuming that, $\varvec{\rm X}(t)$ are observed signals, and $\varvec{S}(t)$ are hidden source signals. In model of linear instantaneous mixed BSS, the relationship between them is linear mixed, i.e.,

3 $$\varvec{X}(t) = \varvec{\rm A} \cdot \varvec{S}(t)$$

$\varvec{\rm A}$ is a N × N dimension constant coefficient matrix. The aim of the BSS is to find a demixing matrix $\varvec{W}$ that is an approximation of the inverse of the original mixing matrix $\varvec{\rm A}$ by repeated iterative calculation according to separation criterion, i.e., $\varvec{W} = \varvec{\rm A}^{{ - \text{1}}}$, and make the output recovering source signals:

4 $$\varvec{U}(t) = \varvec{W} \cdot \varvec{X}(t) = \varvec{S}(t)$$

It needs to be mentioned that, BSS has an inherent uncertainty of orders in outputs.

## Methods

**Fig. 1:**
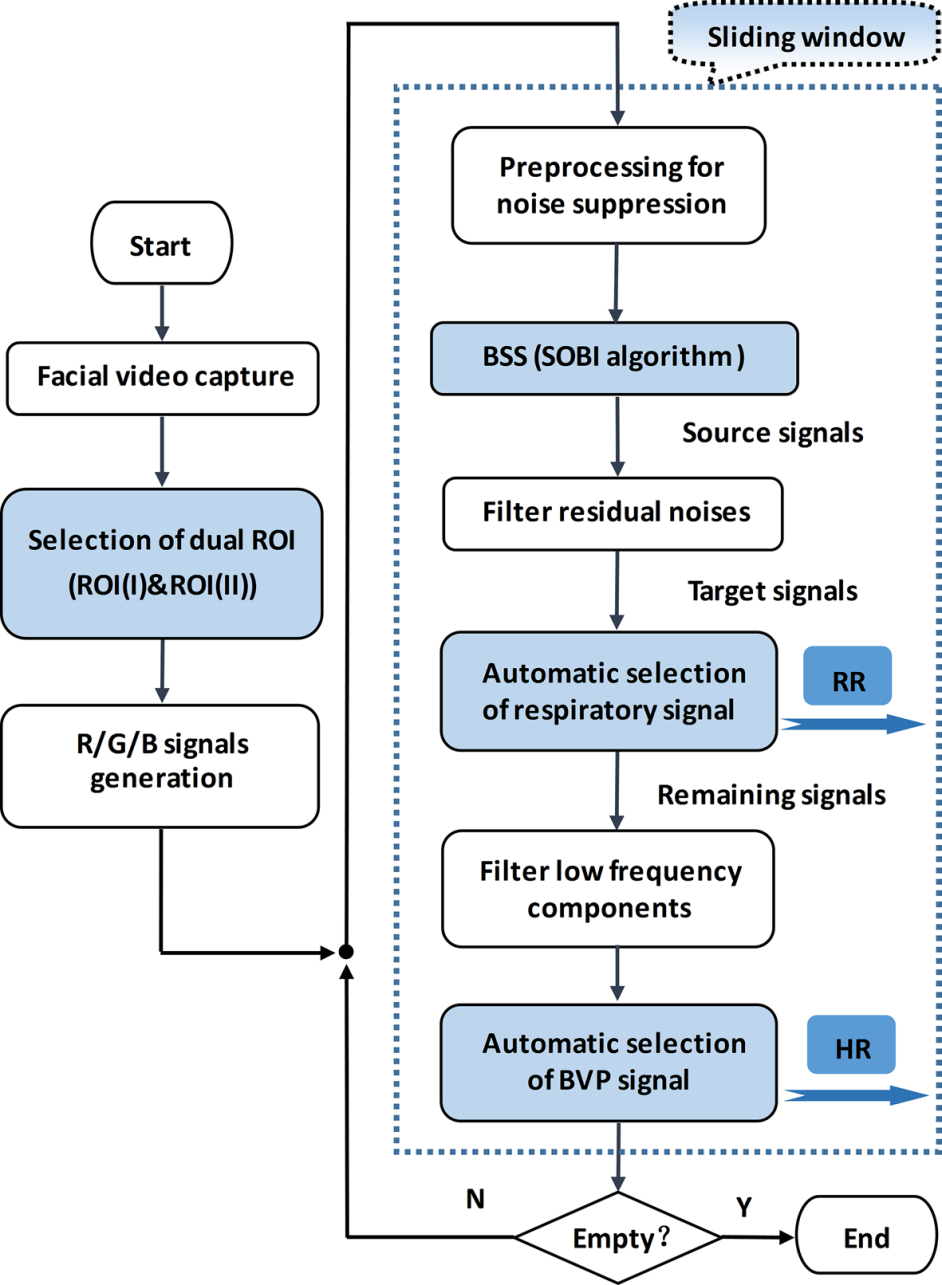
The flow chart of the scheme

In this section, the details of our method are described. The flow chart of the method is shown in Fig. 1. By using front-facing camera of iPhone4s, the facial videos were recorded at a frame rate of 30fps with pixel resolution of 640 × 480 and saved in MOV format for offline analysis on MATLAB2015a platform. For the video, we selected a dual ROI (ROI(**I**)&ROI(**II**)), and calculated the two groups of R/G/B signals based on the dual ROI. After that, we utilized a series of methods and tactics for extracting the RS and BVP signals, then obtained RR and HR. Of note, in the article, all the examples of R/G/B signals were shown based on sliding window. The window length and the sliding step size were set as 600 frames and 150 frames. In spectrum estimation, the length of the FFT was increased from 600 to 2048 by zero filling for increasing the frequency resolution.

### Selection of the dual ROI

**Fig. 2:**
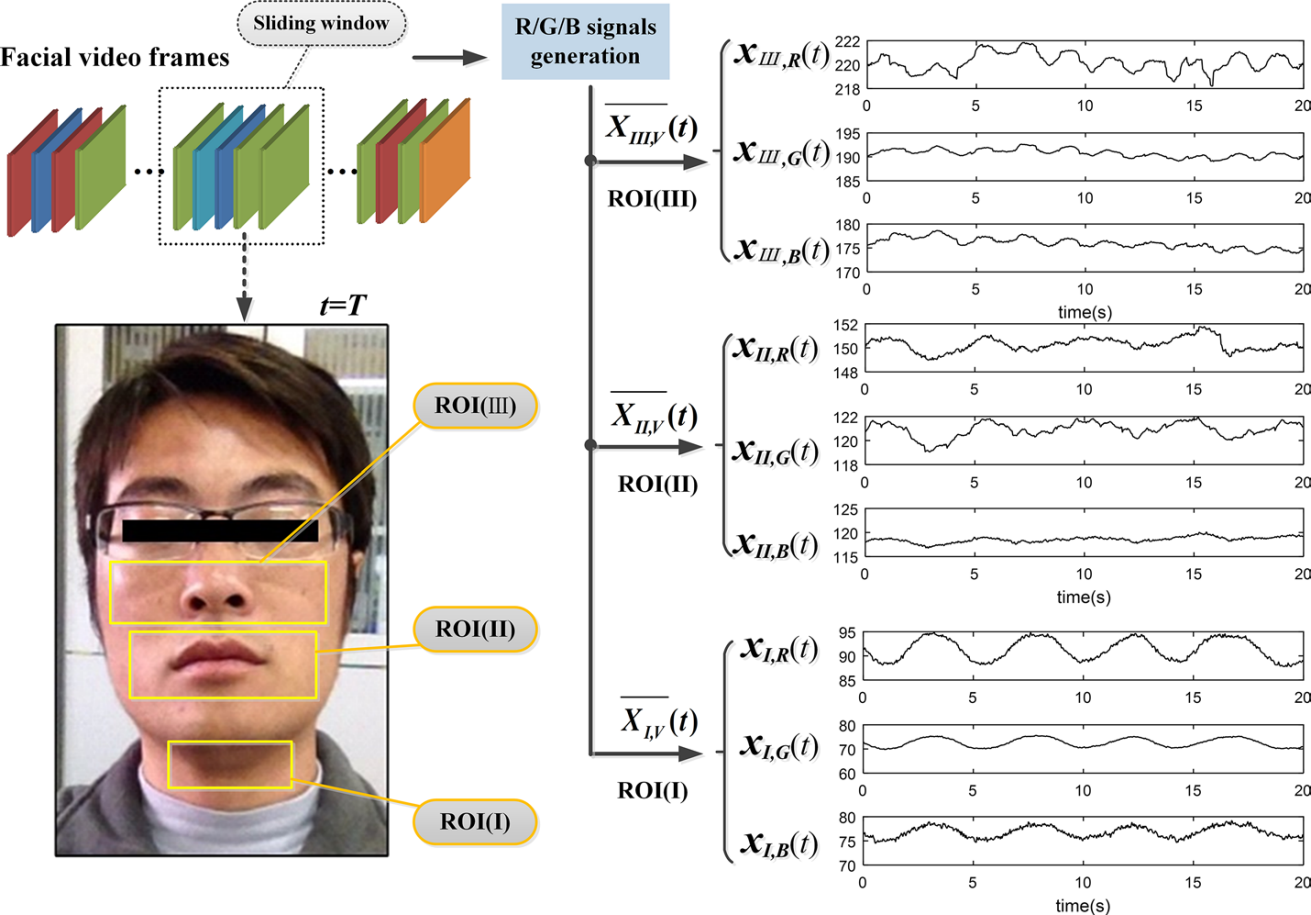
Comparison of R/G/B signals generated based on different ROI

Figure 2 shows the comparison of R/G/B signals calculated based on different sensitive regions. The dual ROI associated with respiration and cardiac pulse is selected according to the comparison.

Most recent literatures have demonstrated that, almost the whole face region could be used for BVP (i.e., HR) measurement [6, 7, 13, 14, 16]. While few practical studies focused on the RS. The process of breathing are often accompanied by the subtle rhythmic movements of some facial organs (such as mouth, nose, neck, etc.), which are commonly treated as motion artifacts. It displays in Fig. 2 that, the distinguished features appear in the waveform of R/G/B signals based on related regions. The throat region (see signals $\overline{{\varvec{X}_{{\varvec{I,V}}} }} (t)$) has the most stable and standard breathing rhythm comparatively, while the mouth region (see signals $\overline{{\varvec{X}_{{\varvec{II,V}}} }} (t)$) appears the feature with a poor stability, and as for the nasal cavity region (see signals $\overline{{\varvec{X}_{{\varvec{III,V}}} }} (t)$), it is inconspicuous. Based on the above analysis, we developed a dual ROI (that is ROI(**I**)&ROI(**II**)) in attempting to obtain synchronous measurement of RR and HR. Of note, the normal fluctuation ranges of RR and HR of the human body are about 12–44 beats/min and 55–140 breath/min, respectively. Therefore, the RR frequency band is set as 0.2–0.8 Hz, and for HR is 0.8–2.3 Hz.

### R/G/B signals preprocessing

After selecting the dual ROI, the facial video will be transformed to two groups of R/G/B signals based on it. It is invariably found that the R/G/B signals are easily contaminated by various noises, including complex motion artifacts, weak ambient light changes, and other complex noises. For the improvement of SNR, three steps, namely high pass filtering (HPF) with cutoff at 0.15 Hz, detrending and normalization, are performed in turn to preprocess the R/G/B signals.

### Selection of BSS algorithm

**Fig. 3:**
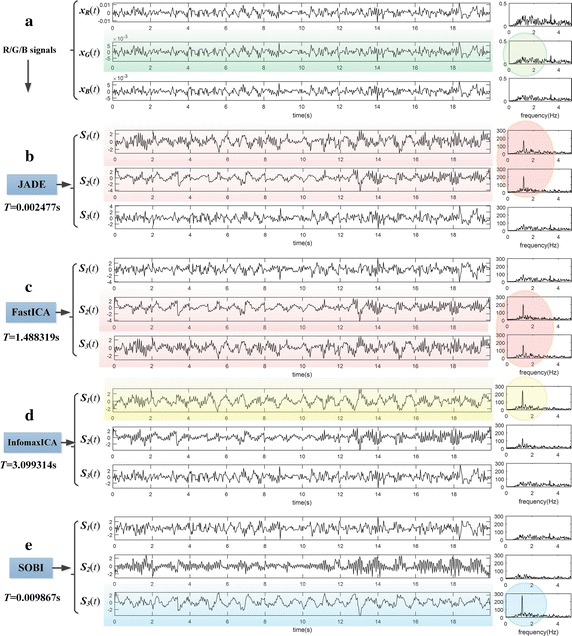
Comparison of separation results using different ICA/BSS methods. **a** There is a segment of R/G/B signals contains less apparent BVP signals in G channel (see the *green circle mark*), **b** after separated by JADE, two ICs contain BVP components emerge in results (see the *red circle mark*), and **c** the situation of FastICA is similar to JADE, **d** furthermore, InfomaxICA algorithm separated out a satisfactory BVP signal (see the *yellow circle mark*), **e** at last, a superior BVP with an outstanding amplitude is obtained by SOBI algorithm (see the *blue circle mark*)

According to our previous experimental results, the SOBI algorithm based on second-order statistics, is superior in performance of R/G/B signals separation and comparatively fairish in computational complexity, compared with other classical ICA/BSS algorithms based on high-order statistics, such as FastICA, InfomaxICA, JADE, etc. Figure 3 is the comparison of separation results on a segment of R/G/B signals selected randomly, which shows the impressive performance of SOBI in R/G/B signals separation. Therefore, in our research, we selected SOBI algorithm for R/G/B signals separation, instead of commonly used JADE or FastICA algorithms.

### Separation of RS and BVP signal

Different from traditional single ROI-based ICA/BSS methods that only extract single target, we explored the dual ROI-based ICA/BSS to separate out the RS and BVP signal from two groups of R/G/B signals. For illustrative purposes, we randomly picked two video segments with different SNR as an example set, and compared the results of two approaches.

#### a. The effect of single ROI-based BSS

We first disposed the example set using traditional single ROI-based BSS approach (i.e., BSS based on 3-channels R/G/B signals). Of note, since each video segment had been transformed to two groups of R/G/B signals based on ROI(**I**) and ROI(**II**) using spatial pixel averaging, it needs twice BSS.

**Fig. 4:**
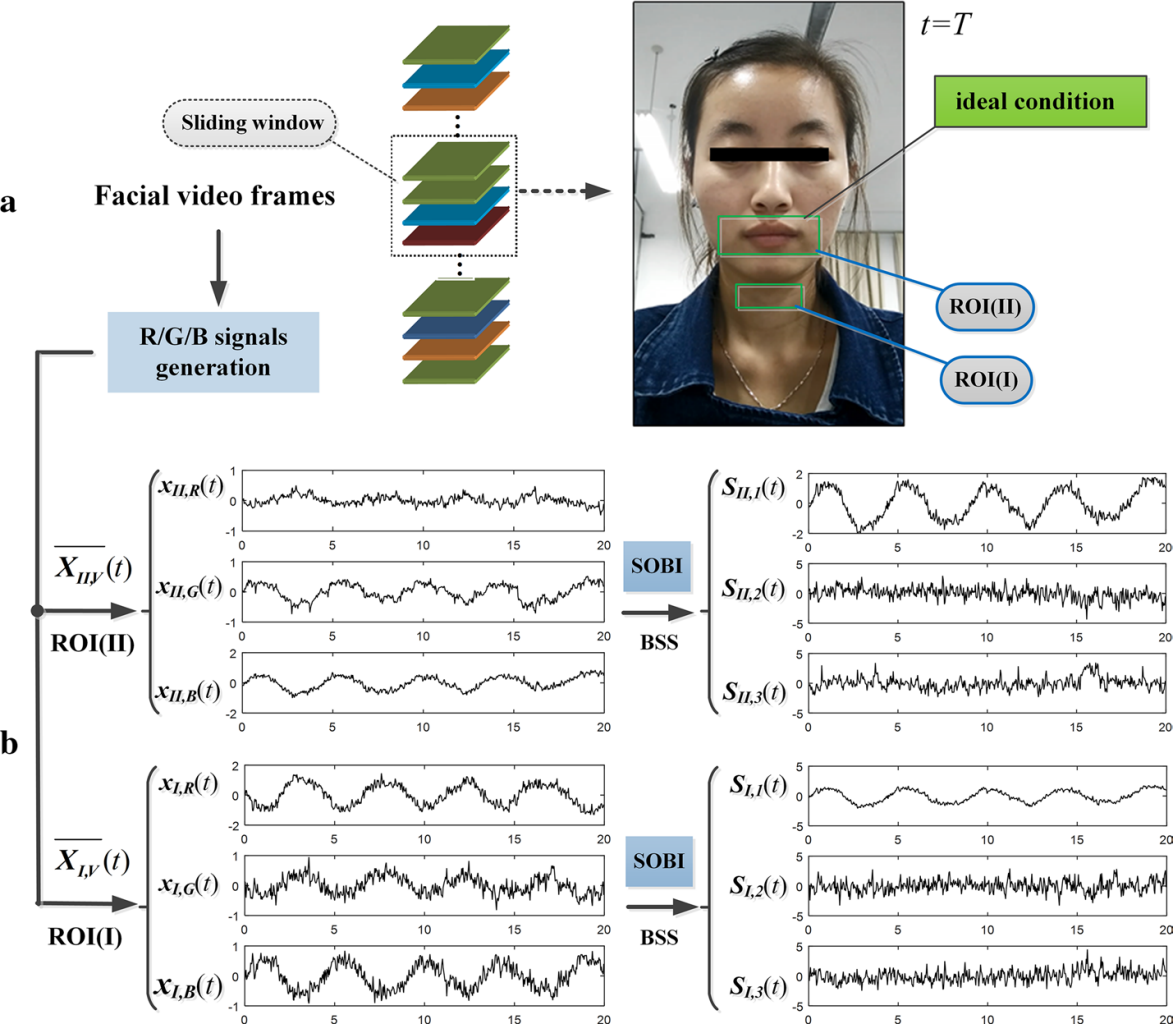
Separation effect based on single ROI for high-quality data: **a** shows the facial video frames captured under ideal condition; **b** displays the two groups of R/G/B signals based on ROI(**I**) and ROI(**II**) and the respective source signals separated by using SOBI algorithm

Figure 4 displays the separation effect of single ROI-based BSS on the first video segment that has a high SNR. It could be observed that, there are clear breathing rhythms in waveform of the two groups of R/G/B signals [see signals [*x*_*I,R*_(*t*), *x*_*I,G*_(*t*), *x*_*I,B*_(*t*)]^*T*^ and [*x*_*II,R*_(*t*), *x*_*II,G*_(*t*), *x*_*II,B*_(*t*)]^*T*^]. After BSS, the breathing rhythms were separated out from the R/G/B signals [see [*S*_*I,1*_(*t*), *S*_*I,2*_(*t*), *S*_*I,3*_(*t*)]^*T*^ and [*S*_*II,1*_(*t*), *S*_*II,2*_(*t*), *S*_*II,3*_(*t*)]^*T*^], while the redundancies still exist on two groups of outputs.

**Fig. 5:**
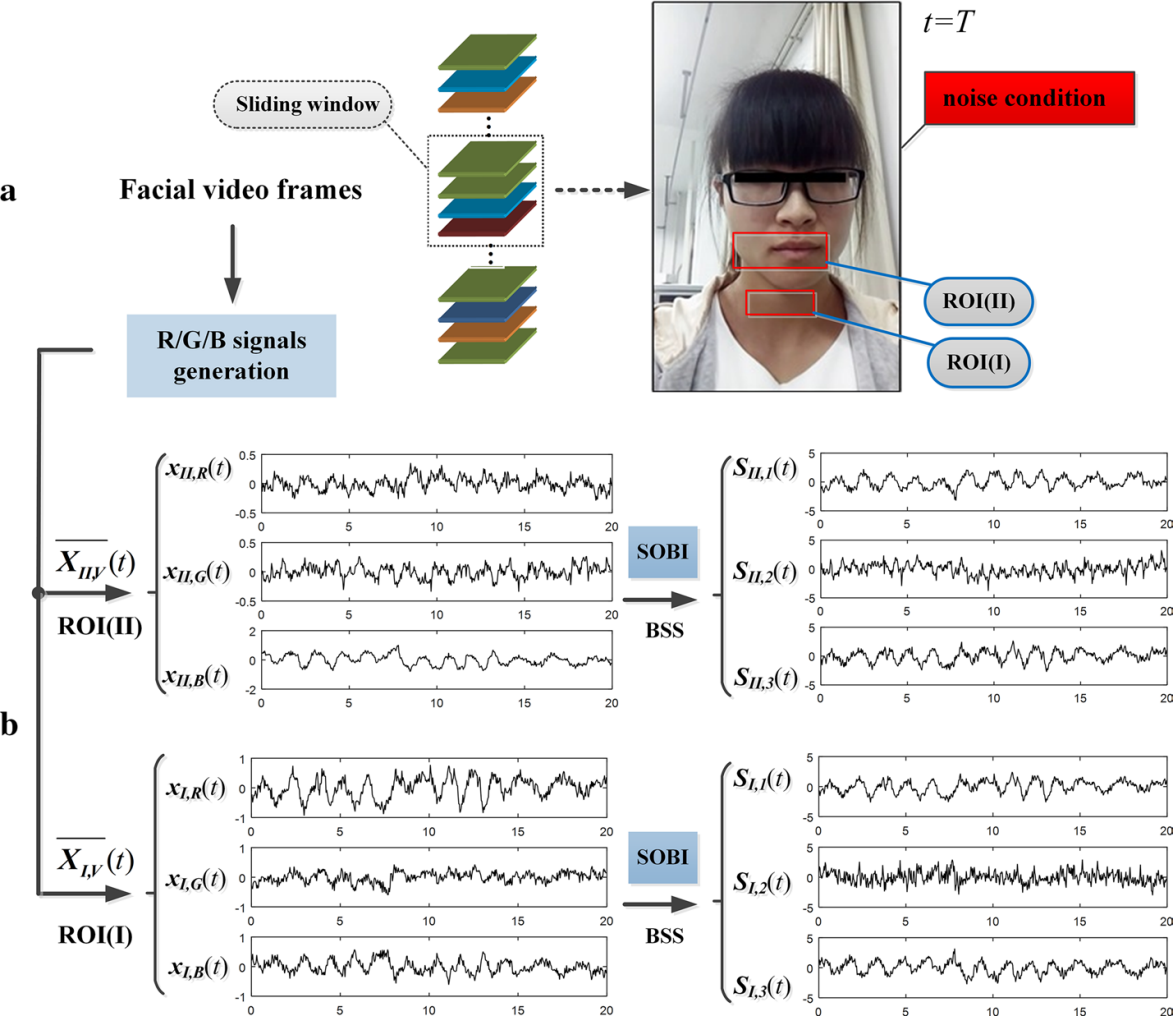
Separation effect based on single ROI for low-quality data: **a** shows the facial video frames captured under noise condition; **b** displays the two groups of R/G/B signals based on ROI(**I**) and ROI(**II**) and the respective source signals separated by using SOBI algorithm

Figure 5 displays the separation effect of single ROI-based BSS on the second video segment that has a low SNR. Being contaminated by complex noises, there is no conspicuous physiological feature appears in waveform of the signals before and after single ROI-based BSS. The separation results are unsatisfactory.

#### b. The effect of dual ROI-based BSS

**Fig. 6:**
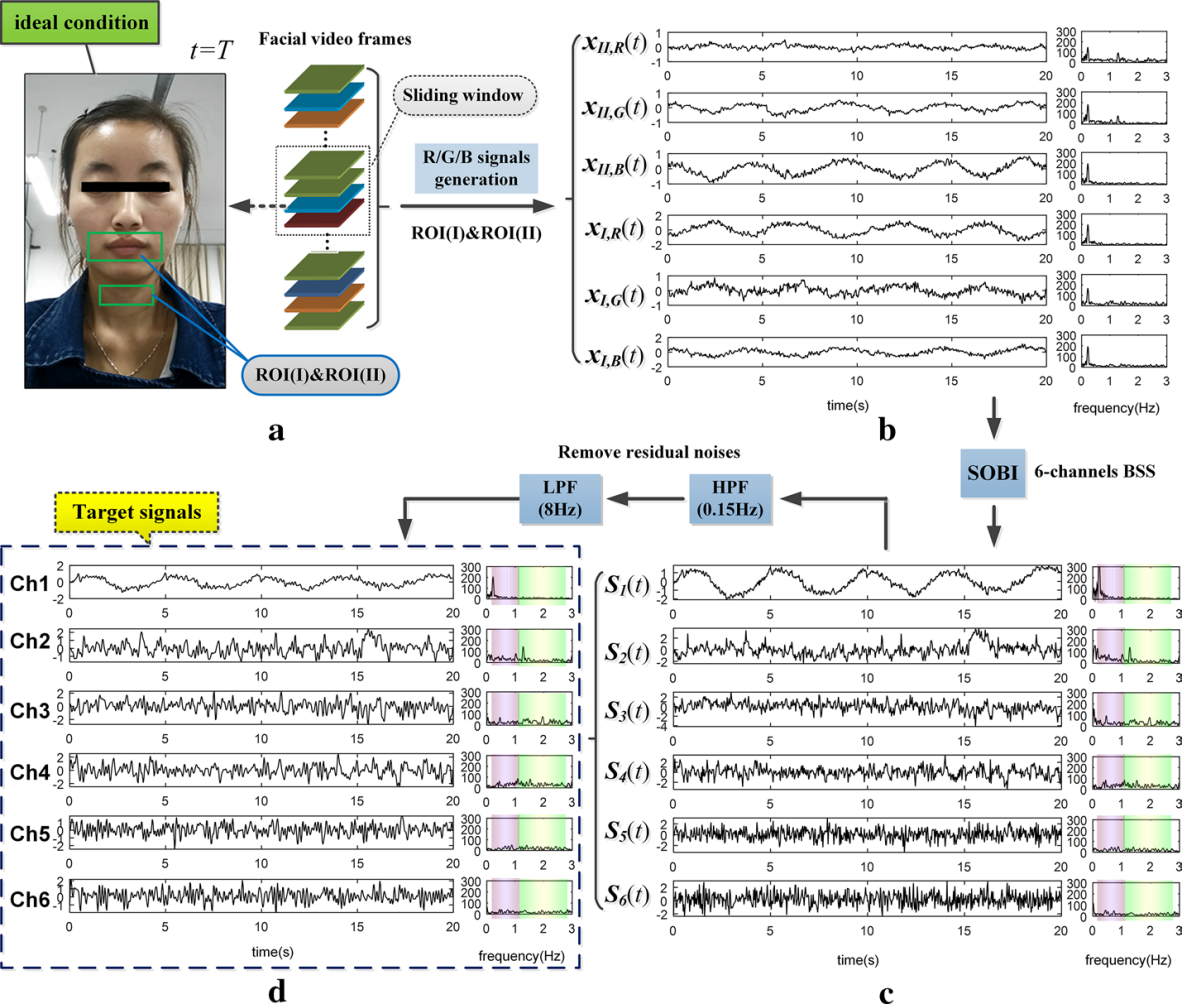
The separation effect of the same data from Fig. 3 by using SOBI based on dual ROI: **a** shows subject’s high-quality video and the dual ROI, then **b** displays 6-channels observations; **c** displays the source signals separated by using 6-channels SOBI; after filters out residual noises, **d** obtains the target signals (note: the *purple* and *green column* on spectrum respectively denote RR band and HR band)

**Fig. 7:**
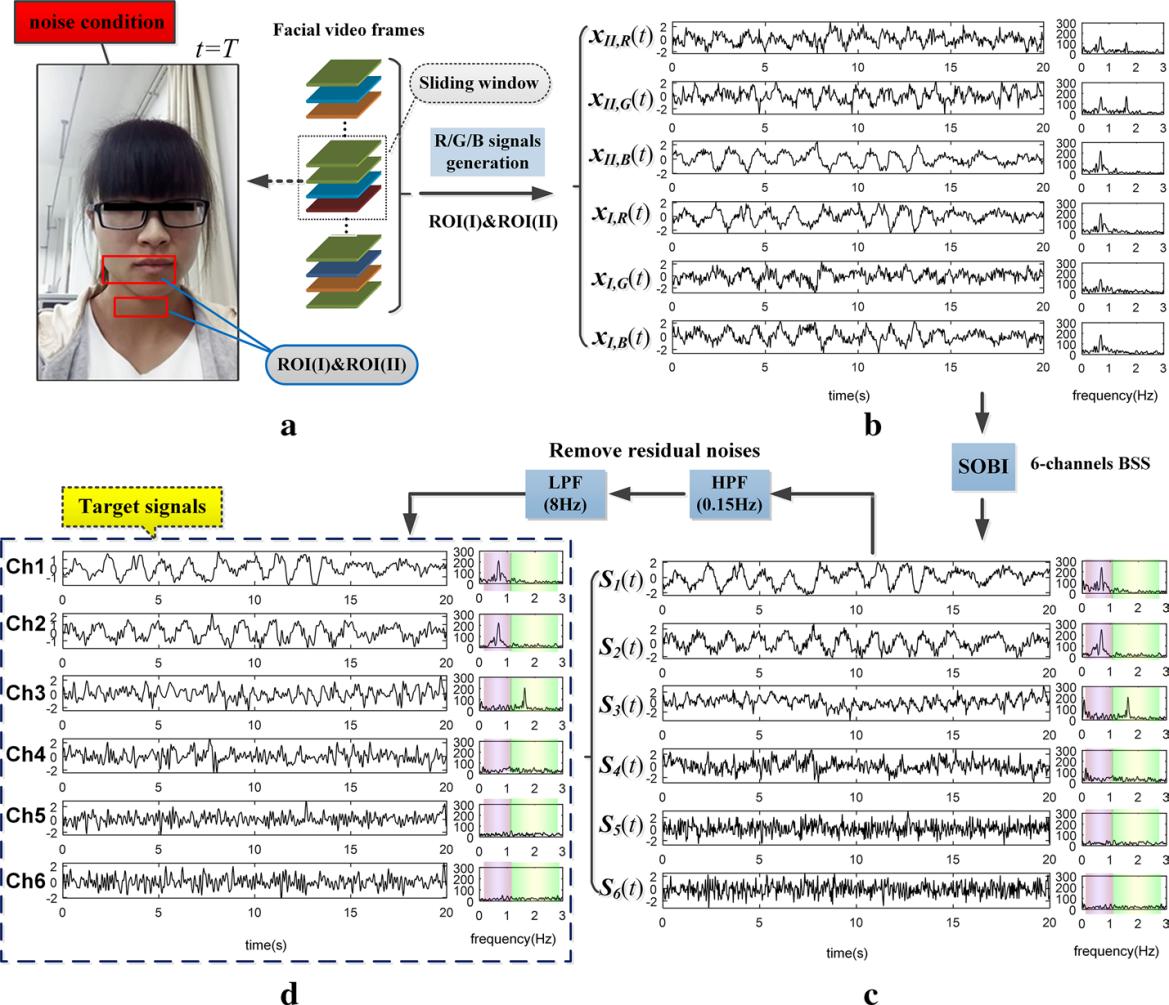
The separation effect of the same data from Fig. 4 by using SOBI based on dual ROI: **a** shows subject’s low-quality video and the dual ROI, then **b** displays 6-channels observations; **c** displays the source signals separated by using 6-channels SOBI; after filters out residual noises, **d** obtains the target signals (note: the *purple* and *green column* on spectrum respectively denote RR band and HR band)

In the above circumstances, the 3-channels BSS based on single ROI is generally insufficient for separating the two physiological signals. The number of observations needs to be increased in order to improve the separation effect. Therefore, we took the two groups of R/G/B signals together as the observations, disposed them by using dual ROI-based BSS (i.e., 6-channels SOBI algorithm). The separation effects of new approach applied on the same two video segments were displayed in Figs. 6 and 7. The two figures show that, the RS and BVP signal were well separated out from the 6-channels R/G/B signals (see Figs. 6c, 7c), by using 6-channels SOBI algorithm.

Nevertheless, there are still some residual noises remained in the source signals (see Fig. 6c: the spectrum obtained by the FFT). We further removed the residual noises by using HPF with cut-off at 0.15 Hz and low pass filtering (LPF) with cut-off at 8 Hz. After the filter processing, the results are defined as the target signals (see Figs. 6d, 7d) that are comparatively clear for further analysis. In Fig. 6d, it can be identified from their spectrum that the Ch1 is RS and Ch2 is BVP signal. While for Fig. 7d, in which Ch3 is BVP signal, yet the RS needs to be further judged on Ch1 and Ch2. For identifying targets in outputs of 6-channels BSS, more automatic selection algorithms are indispensable, especially in presence of low SNR.

### Automatic selections of RS and BVP signal

In our work, we devised the kurtosis-based methods, assisted with some tactics, to achieve the automatic selections of RS and BVP signal.

#### 1. RS selection

**Fig. 8:**
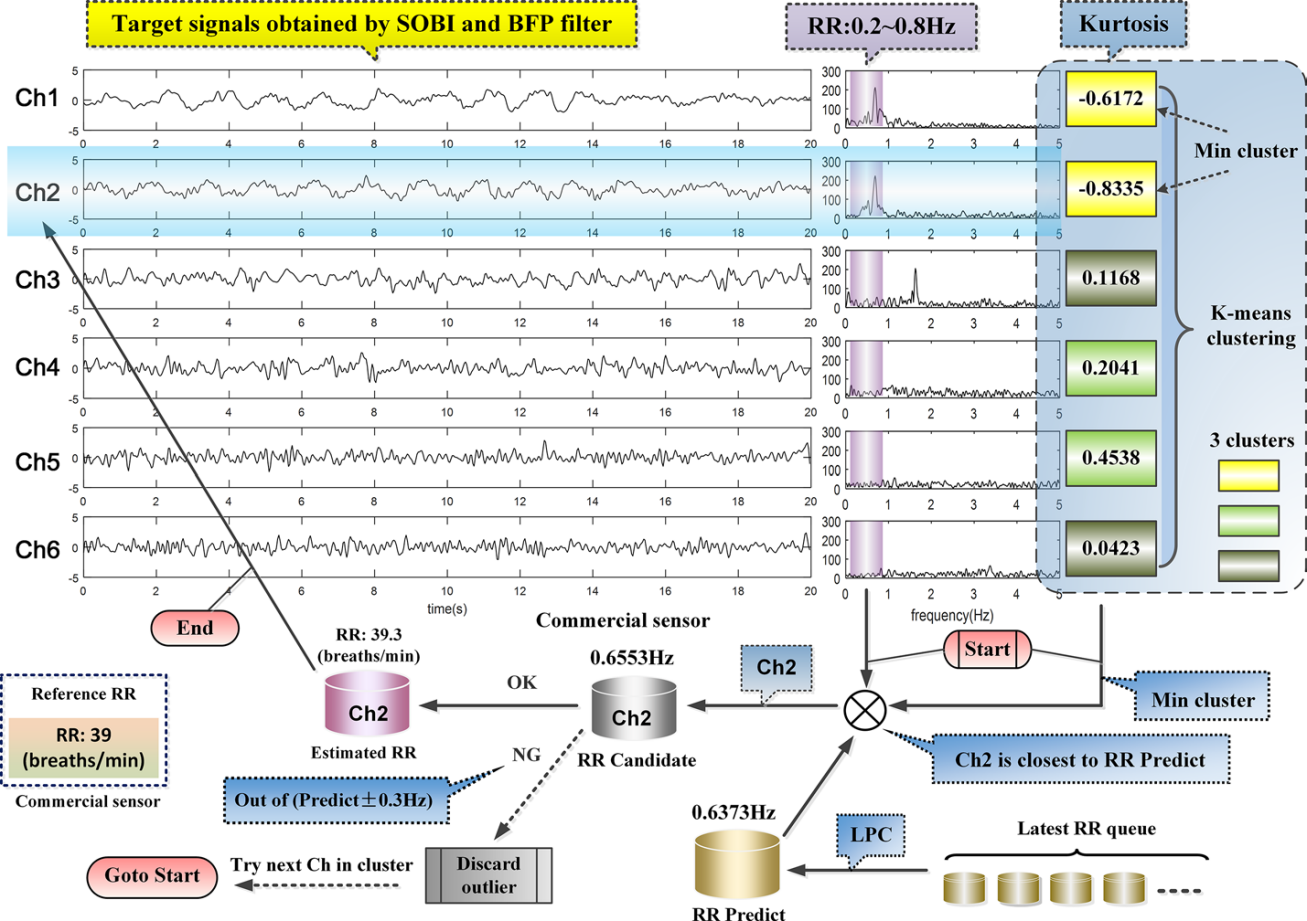
The schematic diagram of automatic selection of the RS

RS could be classified to typical sub-Gaussian signal on account of the feature of the waveform. It might be feasible to identify the RS by measuring sub-Gaussianity of the target signals from the perspective of kurtosis [34]. For data with high SNR as the one in Fig. 6, only the RS belongs to sub-Gaussian signal because of its negative kurtosis, and the largest spectral peak located in RR band is the value of RR desired. While for low SNR data, it is probably the case that several sub-Gaussian components with similar negative kurtosis emerge after BSS, which might interfere with automatic selection of RS. These sub-Gaussian components (low frequency components) might be mainly residual noises remained in RR band (0.2–0.8 Hz) that have not been removed or accidentally results from defective separation. Thus, we utilized some tactics to perfect it. Figure 8 is the schematic diagram of automatic selection of the RS based on the data in Fig. 7d.

It can be seen in Fig. 8 that, there are six channels of target signals and their spectrum marked with purple columns on the RR band (0.2–0.8 Hz) directly from Fig. 7d. Besides, the kurtosis of the target signals are also listed, which were clustered to three clusters (respectively marked with three different colors) by K-means clustering. The minimum cluster is yellow comprised of Ch1 and Ch2 which are all sub-Gaussian signal, with own closed kurtosis. The prediction of the RR was introduced based on the latest five RR values by the linear predictive coding (LPC) method. Then, Ch2 whose largest spectral peak in RR band is closest to the predicted value was selected as the RS candidate. Finally, we confirmed that its spectral peak (RR candidate) was not out of the fluctuation range of the predicted value (±0.3 Hz), then obtained the RS (i.e., Ch2) and RR, otherwise discarded Ch2 as outliers and tried the next one in the minimum cluster.

#### 2. BVP signal selection

After obtaining RS, the five channels target signals remained (it was still six channels if there was no RS identified). In order to avoid interferences from the low frequency components, the HPF with cutoff at 0.8 Hz was used to remove them. Then, the power spectrum kurtosis of the remaining signals were used to detect the BVP components.

**Fig. 9:**
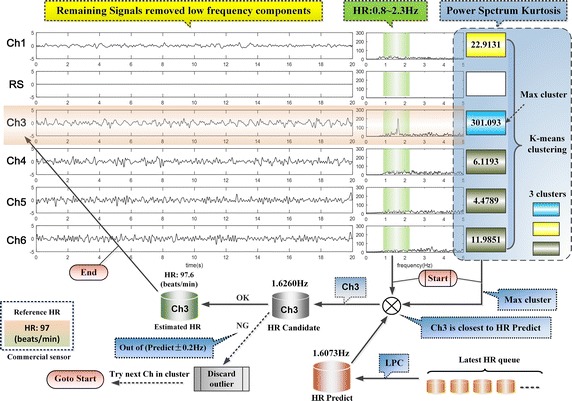
The schematic diagram of automatic selection of the BVP signal

The periodic components would display more distinguishable features on power spectrum kurtosis than power spectrum. In the remaining target signals removed low frequency components, the BVP signal has the strongest periodicity, i.e., the value of power spectrum kurtosis is maximum. Therefore, Power spectrum kurtosis method is feasible to identify the BVP component. Figure 9 is the schematic diagram of automatic selection of the BVP signal based on the five remaining target signals from Fig. 8.

In Fig. 9, there are five channels remaining target signals with low frequency being filtered by HPF (0.8 Hz), the effect of which could be observed in the spectrum that has a green column marked on the HR band (0.8–2.3 Hz). Similar to the Fig. 8 above, we listed the power spectrum kurtosis of the signals, and clustered them to three clusters marked with different colors. The maximum cluster with turquoise color only contained Ch3 as the BVP candidate by chance, and its power spectrum kurtosis value is far greater than others’. Furthermore, we introduced the linear prediction of the HR, which confirmed that the HR candidate was not out of the fluctuation range of the predicted value (±0.2 Hz). Then we obtained the BVP signal (i.e., Ch3) and HR, otherwise discarded Ch3 as outliers. If there is more than one candidate in the maximum cluster, keep on trying until empty.

## Experiments and results

There were eight subjects aged 22–31 years without medical history of heart and respiratory system selected for experiments. The experiments were carried out indoors with adequate and stable ambient light as illumination, according to the experimental paradigms under ideal condition and noise condition. Reference RR and reference HR were recorded by using HKH-11B breathing apparatus and HKG-07A pulse sensor (Hefei Huake Info Technology Co., Ltd.) respectively. For the video recorded, based on sliding window analysis, we obtained estimated RR sequence and HR sequence by the proposed method, without pre-knowledge of the subjects’ actual HR and RR, then compared them with reference values from commercial medical sensors.

### Experiments under ideal condition

We devised the experimental paradigm of ideal condition to acquire data with high SNR for experimental verification. The details are as follows:

1. The subjects need to maintain the condition: sit still without movements, ensuring that face and neck are located in the video region, keeping breaths standard and well-balanced as far as possible.
2. Each subject needs to perform the experiment twice.
3. The time of capturing video in each experiment is limited to 4–6 min, the subject needs to alternate gentle breath (45–60 s) and short breath (45–60 s) at least twice during this time.

Notes: If there are some abrupt movements or jitters happened, which bring serious corruption in commercial medical sensors, the experiment is allowed to be terminated with marking the recording as defective data, and then the subject could give up or try it again after a rest.

There were eight groups of data captured in experiments. We discarded three defective ones, and then obtained a valid original experimental data set with a high SNR. Table 1 shows the level of agreement between the estimated values by the proposed method and reference values from commercial medical sensors. The results of the two methods are strongly correlative from the root-mean-squared error (RMSE) and correlation coefficients.

**Table 1:** Summary of experimental results under ideal condition

Experimental data with high SNR			Statistic (RMSE/correlation coefficient)	
			RR	HR
Group 1	Subject 1	Video 1 (5′03″)	1.51/0.97	1.25/0.96
		Video 2 (4′45″)	1.55/0.96	1.28/0.95
Group 2	Subject 2	Video 3 (4′15″)	2.02/0.90	1.92/0.91
Group 3	Subject 3	Video 6 (4′25″)	1.50/0.97	1.25/0.95
Group 4	Subject 4	Video 7 (4′39″)	1.45/0.98	1.22/0.96
		Video 8 (5′10″)	1.52/0.97	1.32/0.95
Group 5	Subject 5	Video 9 (4′11″)	1.55/0.96	1.14/0.96
		Video 10 (4′35″)	1.49/0.96	0.88/0.98
Group 6	Subject 6	Video 11 (4′55″)	1.50/0.97	1.25/0.96
		Video 12 (5′10″)	1.53/0.96	1.30/0.96
Group 7	Subject 7	Video 13 (5′05″)	1.60/0.95	1.33/0.95
Group 8	Subject 8	Video 15 (5′12″)	1.63/0.96	1.18/0.96
		Video 16 (5′02″)	1.50/0.97	1.21/0.96

RR (breaths/min), HR (beats/min)

**Fig. 10:**
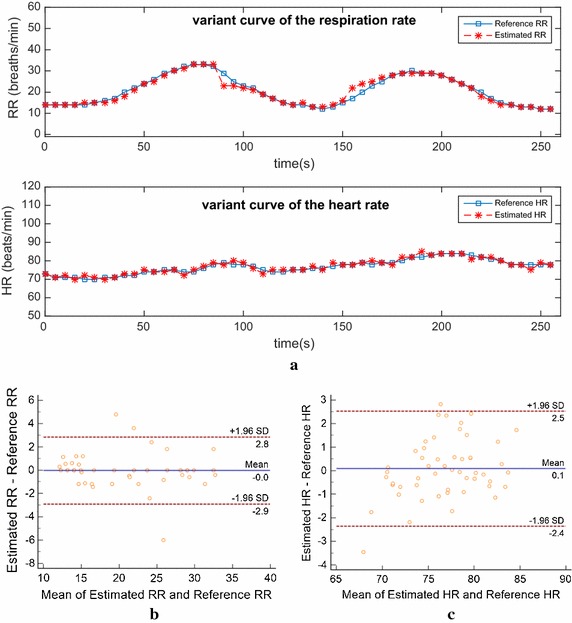
The analysis result on an experimental data under ideal condition. **a** Shows the subject’s variant curves of RR and HR compared with reference values, **b** Bland–Altman plot of the estimated RR against the reference RR, **c** Bland–Altman plot of the estimated HR against the reference HR

For further illustration, we picked out an experimental data (Video 7) from Table 1 for analysis in Fig. 10 (see raw data: Additional files 1, 2, 3). During this experiment, the subject was asked to perform gentle breathing and short breathing alternately twice. It could be observed in Fig. 10a that there are two relatively perfect undulations on the variant curve of RR that effectively reflect the breathing state of the subject throughout the experiment. Furthermore, for RR and HR, the variant curves of the estimated values and reference values are both highly consistent in the waveform. Besides, in Fig. 10b, c, the Bland–Altman plots show that, the mean error (bias) of RR is 0 breaths/min and the 95% confidence interval is [−2.9 2.8], and the parameters for HR are 0.1 beats/min and [−2.4 2.5].

### Experiments under noise condition

Similarly, for acquisition of data with low SNR, the experimental paradigm of noise condition is developed as follows:

1. The subjects maintain the relaxed state: Keep breathing natural and symmetry (some common undesirable conditions are allowed to exist, such as subjects’ subtle involuntary movements, occasional irregular breathing action, swallowing saliva and slight changes in ambient light, etc.).
2. Each subject needs to perform the experiment twice.
3. The time of capturing video in each experiment is limited to 10–15 min, the subject needs to alternate gentle breath (45–60 s) and short breath (45–60 s) about 5–8 times during this time.

Notes: The same as ideal condition mentioned above.

Similar to the procedure above, we reorganized experimental data with discarding the defective one. Then, the result of the statistic was given in Table 2. It indicates that the measurements by the proposed method are closely correlative to reference values under noise condition.

**Table 2:** Summary of experimental results under noise condition

Experimental data with low SNR			Statistic (RMSE/correlation coefficient)	
			RR	HR
Group 1	Subject 4	Video 1 (12′03″)	2.52/0.92	1.90/0.89
		Video 2 (10′01″)	2.15/0.97	2.32/0.83
Group 2	Subject 2	Video 3 (13′25″)	2.83/0.82	2.91/0.81
Group 3	Subject 3	Video 5 (12′30″)	2.63/0.89	1.91/0.91
		Video 6 (12′55″)	2.25/0.91	2.12/0.89
Group 4	Subject 8	Video 7 (11′10″)	2.45/0.92	1.82/0.93
		Video 8 (11′36″)	2.58/0.91	1.98/0.93
Group 5	Subject 1	Video 9 (12′20″)	2.62/0.87	1.70/0.95
		Video 10 (12′58″)	2.43/0.88	1.64/0.93
Group 6	Subject 6	Video 11 (13′01″)	2.48/0.89	1.90/0.89
		Video 12 (13′59″)	3.15/0.80	1.65/0.91
Group 7	Subject 7	Video 13 (12′11″)	2.18/0.92	1.81/0.92
		Video 14 (12′29″)	2.28/0.89	1.95/0.90
Group 8	Subject 5	Video 15 (13′07″)	2.08/0.91	1.69/0.93
		Video 16 (12′55″)	2.55/0.86	1.92/0.90

RR (breaths/min), HR (beats/min)

**Fig. 11:**
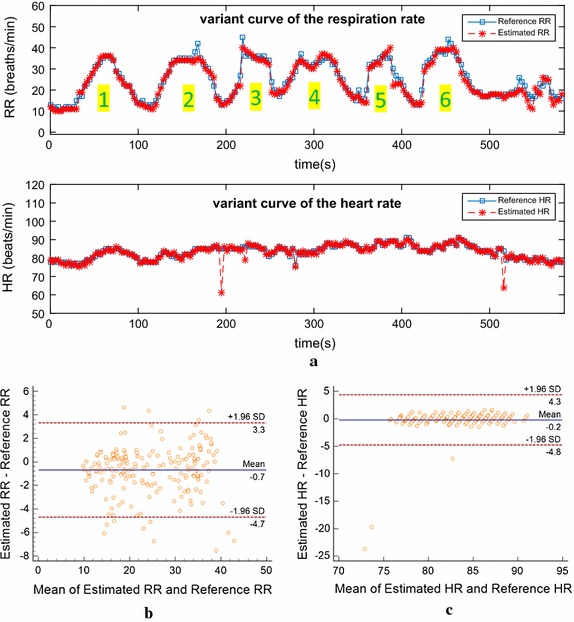
The analysis result on an experimental data under noise condition. **a** Shows the subject’s variant curves of RR and HR compared with reference values, **b** Bland–Altman plot of the estimated RR against the reference RR, **c** Bland–Altman plot of the estimated HR against the reference HR

Figure 11 shows the analysis results on the Video2 picked out from Table 2 (see raw data: Additional files 4, 5, 6). During this experiment, the subject was asked to alternate gentle breathing and short breathing six times. It could be observed that, there are six undulations on the subject’s variant curve of estimated RR. Although the waveform of estimated RR sequence is not perfect, it is basically consistent with the reference data, and roughly reflects the subject’s breathing state. Moreover, the subject’s estimated HR sequence is impressively stable and less affected.

## Discussions

**Fig. 12:**
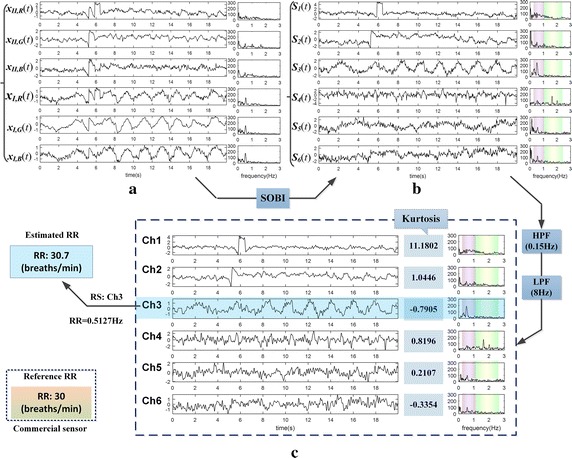
The separation and RS automatic selections results on a random low SNR data: **a** shows a segment of 6-channel R/G/B signals with low SNR, then separated by 6-channels SOBI, **b** displays the source signals, after filters out residual noises, **c** obtains the target signals. By kurtosis-based method, the RS (Ch3) was detected accurately, with estimated RR being approximate to reference value from commercial sensor

We extracted RS and BVP signals from the face video synchronously by BSS, and achieved dynamic variations of RR and HR that were good in agreement with commercial sensors. Although many researches have mentioned the estimations of several physiological parameters, these estimations are mainly relying on sophisticated video tracking and detection algorithms for motion artifact attenuation. However, our research manifested that, instead of video processing algorithms, the ICA/BSS approaches could appropriately separate out BVP signals, motion artifacts and other noises. Moreover, we creatively obtained RS based upon the rhythmic respiratory motion artifacts. We also carried out some optimization or explorations in ICA/BSS-based IPPG techniques as follow:

1. *Dual ROI-based BSS* For the insufficient capability of separation in single ROI-based ICA/BSS (see Figs. 4b, 5b), we explored the potential of dual ROI-based BSS. The dual ROI comprised of throat region (ROI(I)) and mouse region (ROI(II)) were selected based on experimental analysis (see Fig. 2). By applying BSS on 6-channel R/G/B signals yielded from dual ROI, we separated out RS (i.e., respiratory motion artifacts) and BVP signal adequately. It is worth noting that, the throat region (ROI(I)), commonly exposed in facial video, with stable and standard breathing rhythm, might be suitable for practical breath detection.
2. *SOBI algorithm* To separate out the target signals, the existed ICA/BSS-based IPPG approaches commonly utilized classical ICA algorithms based on higher-order statistics, such as JADE or FastICA algorithms, yet the performances were neglected. In our research, we selected SOBI algorithm instead, which is superior in performance of R/G/B signals separation and good in computational complexity. These superiorities might guarantee the proposed method more potential for applications on different platforms, for instance, the smart phone.
3. *Kurtosis-based methods for automatic selection* Based upon analysis of the statistical characteristics of RS and BVP signals, we devised the kurtosis-based method and power spectrum kurtosis-based method respectively for reliable automatic selections. Of note, under low SNR situations, defective separation might accidentally emerge, because the separation of BSS is contaminated by complex noises (see Fig. 7c). In Fig. 7c, it encountered an unexpected case that the two ICs (Ch1and Ch2) are closed on waveform or spectrum. To our knowledge, they both belong to the same RS. Nevertheless, according to the RS automatic selections method, Ch2 was detected as RS (see Fig. 8), and the accuracy could still be maintained. In our practical tests, under low SNR situations, it is commonly the case that 6-channel R/G/B signals could be separated well by ICA/BSS algorithms, with accurate selections of targets. Figure 12 shows the separation and RS automatic selections results on a segment of 6-channel R/G/B signals with low SNR selected randomly.

Moreover, in the proposed method, the bands of RR and HR were set as 0.2–0.8 and 0.8–2.3 Hz respectively. For purpose of noises removal, we utilized different filters, and there are several reasons behind them as below:

First of all, in the preprocessing, we carried out HPF for removing the intricate low-frequency noises in R/G/B signals, where the cut-off frequency of filter was set as 0.15 Hz, which is an adapted value through adjustment. Considering the requirement of ICA/BSS in mechanism that observations should retain the statistical data (especially high frequency components) as much as possible, so we gave up LPF that is the removal of high-frequency noises.

Furthermore, after BSS, it is found that quite a few residual noises emerged in ICs, which would interfere with automatic selections (see Figs. 5c, 6c). Hence, we took measures to filter them. The filter is set as HPF (0.15 Hz) and LPF (8 Hz) for the following reasons: Firstly, the residual low-frequency noises with sub-Gaussianity would cause misjudgment in RS automatic selections. So we performed the HPF (0.15 Hz) that is beneficial for resolving the problem. Secondly, the residual high-frequency noises would bring a measurable impact on the kurtosis of ICs, thus it is essential to depress the noises by applying LHF. However, it is meaningful that attention is required to maintain non-destructive BVP signals in processing, for further researches such as HRV, etc. In consideration of the frequency band of BVP (0.8–2.3 Hz), an excessively low cut-off frequency of LHF is inappropriate for preserving the 2nd and 3rd harmonic components of BVP. Consequently, we selected LPF (8 Hz) by practical test.

The last but not the least, the automatic selection of BVP signal depends on the strong periodicity of BVP, while the essential condition is to remove the out-of-band noises, especially the lower frequency noises as clean as possible. Consequently, after detecting RS, we further filtered the low-frequency band by using HPF (0.8 Hz), to avoid interference from unknown periodic components.

**Fig. 13:**
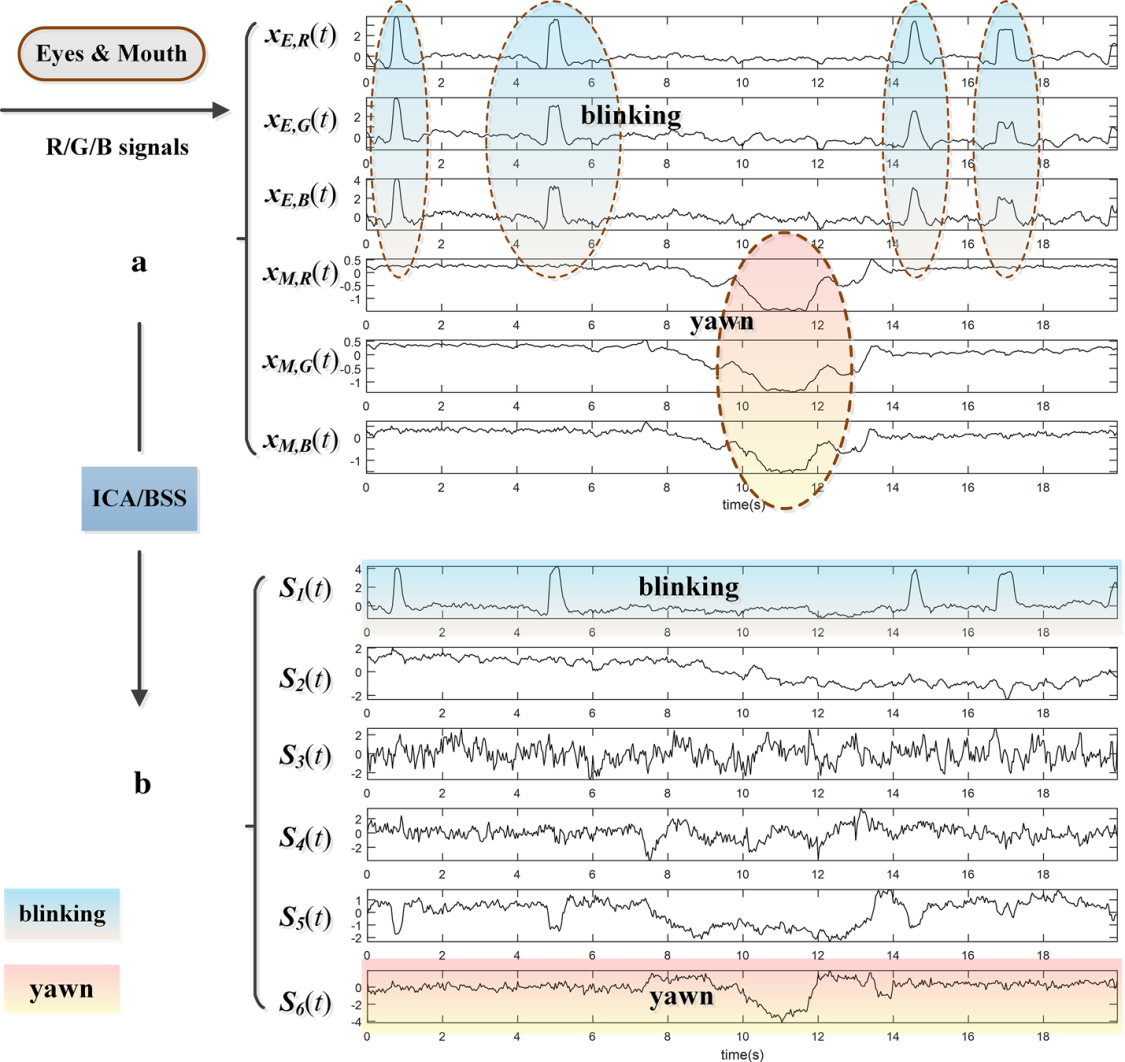
The test of blinking and yawn signs extraction based upon motion artifacts: **a** shows a segment of 6-channel R/G/B signals yielded from regions of eyes and mouth respectively, in which could be seen apparent motion artifacts caused by blinking and yawn. Through ICA/BSS, **b** displays that the blinking and yawn are extracted appropriately as physiological signs in the separation results

Besides, it is significant to be mentioned that the proposed method has the potentials for extracting more vital signs, such as blinking, wrinkling nose, yawn, as well as other muscular movements, which are all intricate local motion artifacts for facial video tracking and detection algorithms. Figure 13 demonstrates that blinking and yawn signs could be extracted from relevant local facial regions. Thus, it is easy to understand that the idea of the proposed method might also be applied to other IPPG-based applications such as emotion computation and fatigue detection, etc.

There are still some limitations existed in the proposed method. In our research, we have not employed facial video tracking and automatic region selection algorithms, which brought a shortcoming that the ROI can only be marked manually. Besides, the proposed method might be unable to handle the serious artifacts caused by heavy motions, which gave rise to serious drifts or deformation on R/G/B signals (the problem also exists in clinical applications of many commercial medical sensors). We are taking efforts to cover these problems.

## Conclusion

Dynamic measurements of RR and HR from facial video have been proved in this proposed method. And it may have the potentials to be extended further to extract more physiological parameters, such as HRV, eye blinking, wrinkling nose, yawn, and other muscular movements, etc. The research has a good application prospect in the field of face-based physiological parameters assessments or emotion computation fields, especially for the subjects in the trial, the sniper or the people under special working environment. All the related issues could have further exploration in follow-up work.

## Additional files

**Additional file 1.** The raw video picked from Table 1 for analysis in Fig. 10.

**Additional file 2.** Reference RR recorded by commercial medical sensors during recording video 7.

**Additional file 3.** Reference HR recorded by commercial medical sensors during recording video 7.

**Additional file 4.** The raw video picked from Table 2 for analysis in Fig. 11.

**Additional file 5.** Reference RR recorded by commercial medical sensors during recording video 2.

**Additional file 6.** Reference HR recorded by commercial medical sensors during recording video 2.

## Abbreviations

IPPG: imaging photoplethysmography
HR: heart rate
RR: respiratory rate
BSS: blind source separation
ROI: region of interest
R/G/B: Red/Green/Blue
SOBI: second-order blind identification
RS: respiratory signal
BVP: blood volume pulse
HRV: heart rate variability
ICA: independent component analysis
JADE: joint approximate diagonalization of eigenmatrices
VJ: Viola-Jones
SURF: speeded-up robust features
SNR: signal-to-noise ratio
HPF: high pass filtering
LPF: low pass filtering
LPC: linear predictive coding
RMSE: root-mean-squared error

## References

[1] Wu T (2000). Photoplethysmography imaging: a new noninvasive and non-contact method for mapping of the dermal perfusion changes. Proc SPIE.

[2] Lee J, et al. Comparison between red, green and blue light reflection photoplethysmography for heart rate monitoring during motion. In: Proceedings Conference of the IEEE engineering in medicine and biology society; 2013. p. 1724–7. 10.1109/EMBC.2013.6609852 24110039

[3] Sun Y (2012). Use of ambient light in remote photoplethysmographic systems: comparison between a high-performance camera and a lowcost webcam. J Biomed Opt.

[4] Hu SJ, et al. Feasibility of imaging photoplethysmography. In: Proceedings of international conference on biomedical engineering; 2008. p. 72–5.

[5] Hu S, et al. Development of effective photoplethysmographic measurement techniques: from contact to non-contact and from point to imaging. In: Proceedings conference of the IEEE engineering in medicine and biology society, vol 2009; 2009. p. 6550–3. 10.1109/IEMBS.2009.5334505 19964902

[6] Takano C, Ohta Y (2007). Heart rate measurement based on a time-lapse image. Med Eng Phys.

[7] Verkruysse W (2008). Remote plethysmographic imaging using ambient light. Opt Exp.

[8] Scully CG (2012). Physiological parameter monitoring from optical recordings with a mobile phone. IEEE Trans Biomed Eng.

[9] Jonathan E, Leahy MJ (2011). Cellular phone-based photoplethysmographic imaging. J. Biophoton.

[10] Jonathan E, Leahy M (2010). Investigating a smartphone imaging unit for photoplethysmography. Physiol Meas.

[11] Matsumura K (2014). iPhone 4s photoplethysmography: which light color yields the most accurate heart rate and normalized pulse volume using the iphysiometer application in the presence of motion artifact. PLoS ONE.

[12] Hayes MJ, Smith PR (1998). Artifact reduction in photoplethysmography. Appl Opt.

[13] Poh MZ (2011). Advancements in noncontact, multiparameter physiological measurements using a webcam. IEEE Trans Biomed Eng.

[14] Poh MZ (2010). Non-contact, automated cardiac pulse measurements using video imaging and blind source separation. Opt Exp.

[15] Lewandowska M, Nowak J (2012). Measuring pulse rate with a webcam. J Med Imag Health Inform.

[16] Sun Y (2011). Motion-compensated noncontact imaging photoplethysmography to monitor cardiorespiratory status during exercise. J Biomed Opt.

[17] Lewandowska M, et al. Measuring pulse rate with a webcam: a non-contact method for evaluating cardiac activity. In: Proceedings of the FedCSIS, Szczecin, Poland; 2011. p. 405–10.

[18] Tsouri G, Kyal S, Dianat S, Mestha L (2012). Constrained independent component analysis approach to nonobtrusive pulse rate measurements. J Biomed Opt.

[19] McDuff D, Gontarek S, Picard R (2014). Remote detection of photoplethysmographic systolic and diastolic peaks using a digital camera. IEEE Trans Biomed Eng.

[20] Wu HY (2012). Eulerian video magnification for revealing subtle changes in the world. ACM Trans Graph.

[21] Hertzman AB, Spealman C (1937). Observations on the finger volume pulse recorded photoelectrically. Amer J Physiol.

[22] Sayers B (1973). Analysis of heart rate variability. Ergonomics.

[23] Sun Y, Thakor N (2016). Photoplethysmography revisited: from contact to noncontact from point to imaging. IEEE Trans Biomed Eng.

[24] Wang W (2015). Exploiting spatial redundancy of image sensor for motion robust rPPG. IEEE Trans Biomed Eng.

[25] Henriques JF, et al. Exploiting the circulant structure of tracking by-detection with kernels. In: Proceedings of European conference on computer vision, vol 7575; 2012. p. 702–15.

[26] Feng L (2015). Motion-resistant remote imaging photoplethysmography based on the optical properties of skin. IEEE Trans Circuits Syst Video Technol.

[27] Emrah Tasli H, Gudi A, Uyl M. Remote PPG based vital sign measurement using adaptive facial regions. IEEE International Conference on Image Processing; 2014.

[28] Kumar M (2015). Distance PPG: robust non-contact vital signs monitoring using a camera. Biomed Opt Exp.

[29] Blackford EB, Estepp JR (2015). Effects of frame rate and image resolution on pulse rate measured using multiple camera imaging photoplethysmography. Proc SPIE.

[30] Estepp JR, et al. Recovering pulse rate during motion artifact with a multi-imager array for non-contact imaging photoplethysmography. In: Proceedings IEEE international conference on systems, man and cybernetics; 2014, p. 1462–9.

[31] Holton B (2013). Signal recovery in imaging photoplethysmography. Physiol Meas.

[32] Kwon S, et al. Validation of heart rate extraction using video imaging on a built-in camera system of a smartphone. In: Proceedings of 34th IEEE EMBS, San Diego; 2012. p. 2174–7. 10.1109/EMBC.2012.6346392 23366353

[33] de Haan G, Jeanne V (2013). Robust pulse-rate from chrominance-based rPPG. IEEE Trans Biomed Eng.

[34] Shen L, et al. Human detection based on the excess kurtosis in the non-stationary clutter environment using UWB impulse radar. International Asia-Pacific conference on Synthetic Aperture Radar (APSAR), Seoul; 2011. p. 1–4.

